# Supplementary figures and images for: Overlooked Candida glabrata petites are echinocandin tolerant, induce host inflammatory responses, and display poor in vivo fitness
Source: mBio. 2023 Sep 29;14(5):e01180-23. doi: 10.1128/mbio.01180-23 (PMC10653939; doi:10.1128/mbio.01180-23)

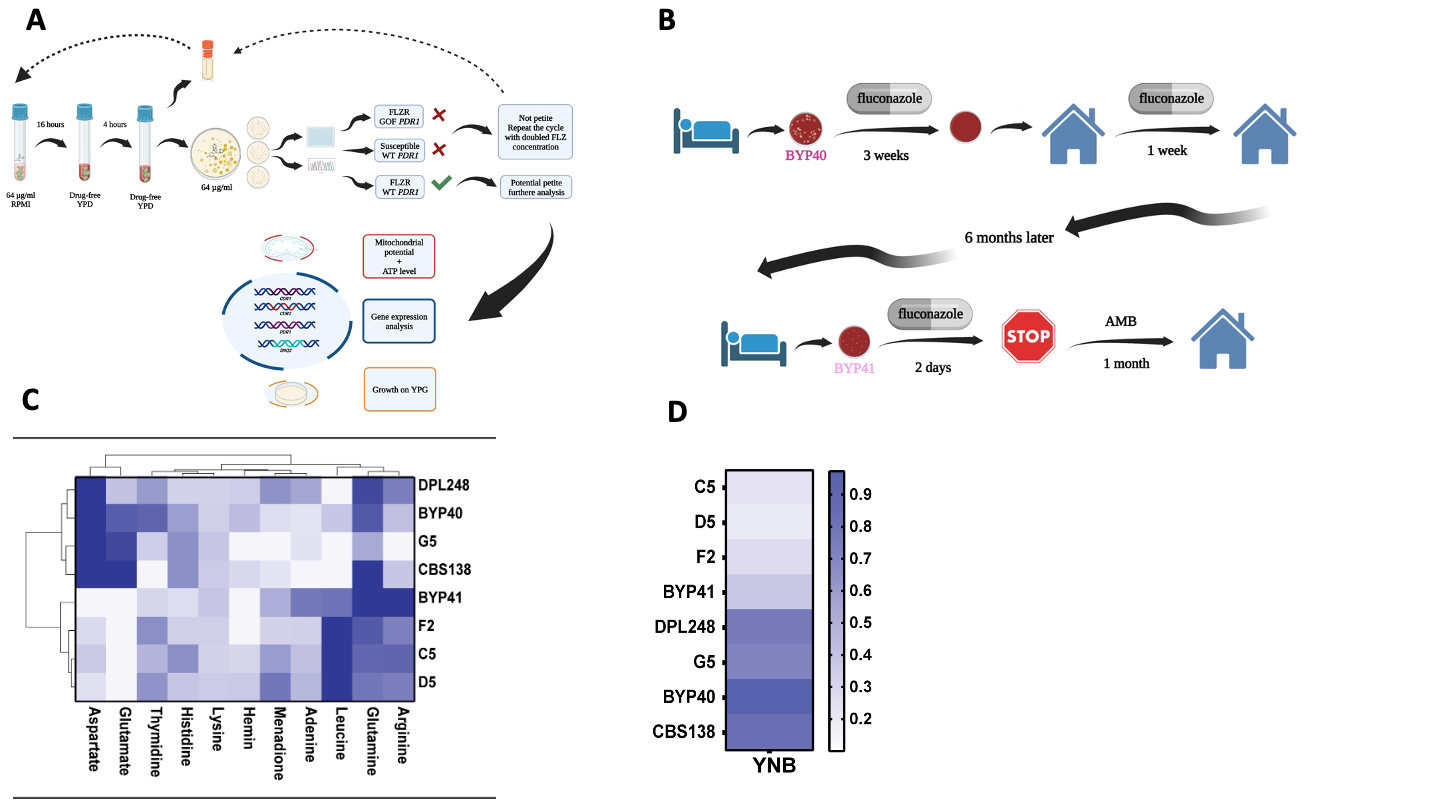

Supplement: Figure S1 — Processes involved in the development of laboratory and clinically derived petite C. glabrata isolates. [file mbio.01180-23-s0001.tif]

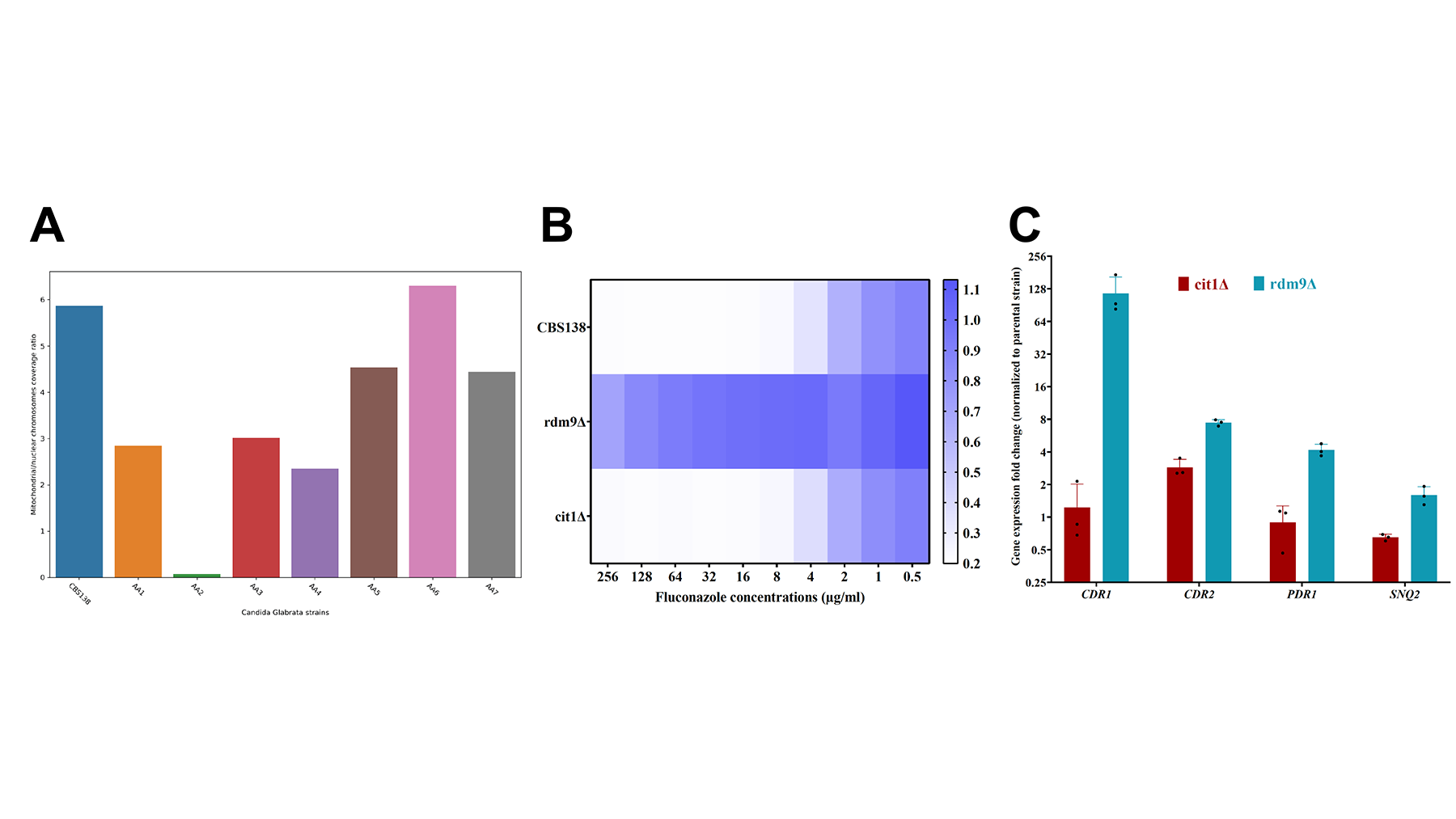

Supplement: Figure S2 — Mitochondrial DNA (mtDNA) coverage of petite and non-petite isolates using whole-genome sequencing identified that petite isolates have a lower mtDNA than non-petites. [file mbio.01180-23-s0002.tif]

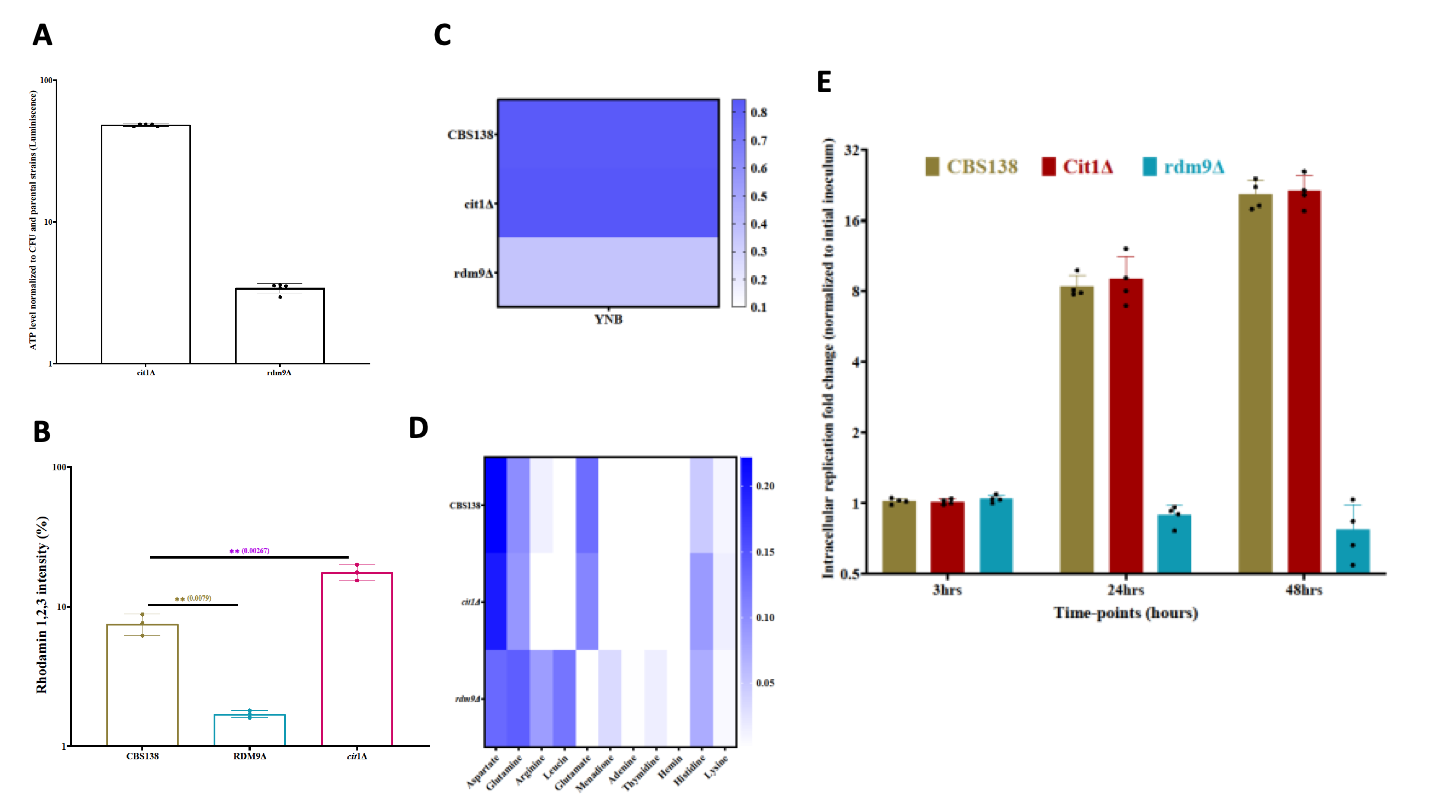

Supplement: Figure S3 — Similar to petite isolates, Rdm9Δ has a significantly lower level of ATP than the parental strain CBS138. [file mbio.01180-23-s0003.tif]

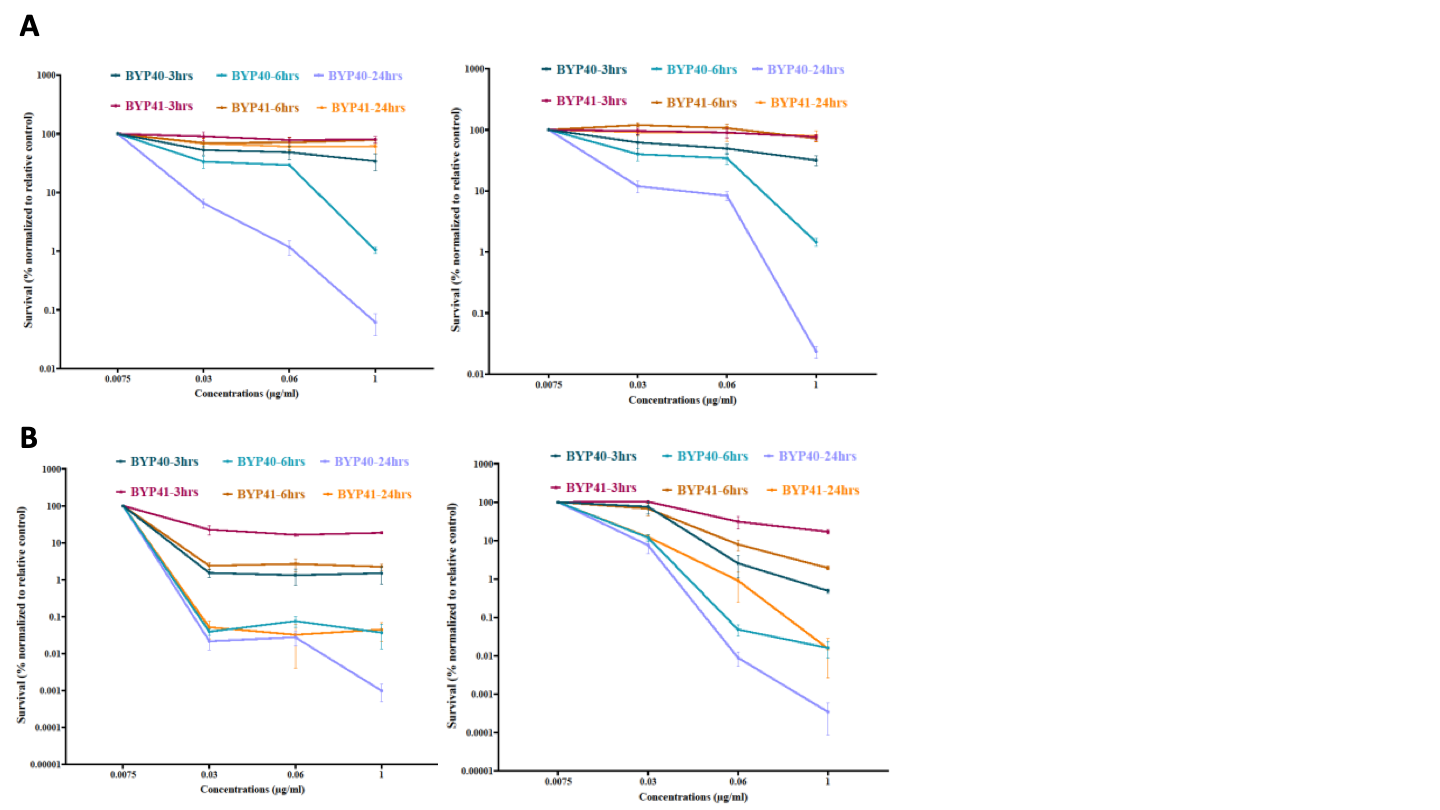

Supplement: Figure S4 — Non-responsiveness of intracellular petites to echinocandins. [file mbio.01180-23-s0004.tif]

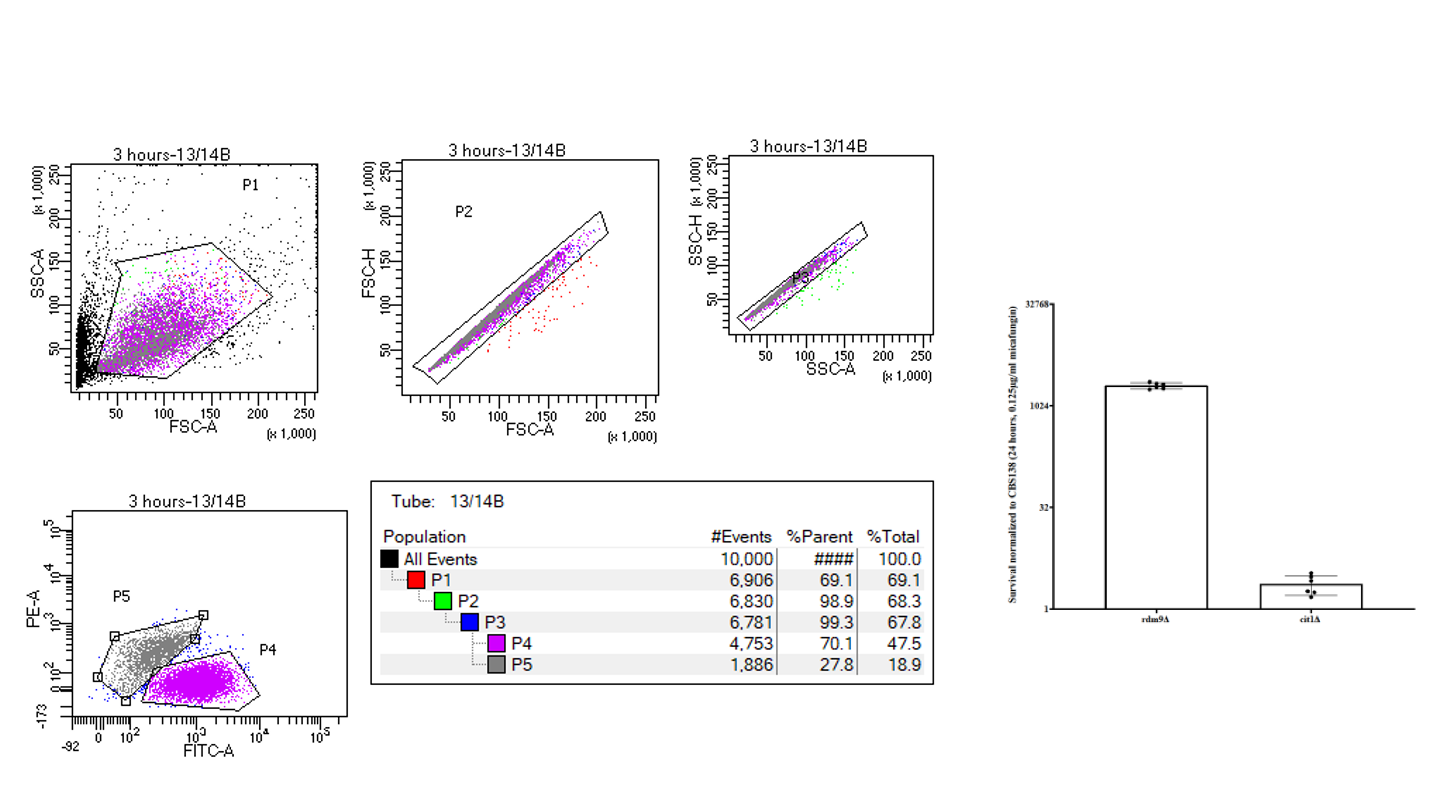

Supplement: Figure S5 — Flow cytometry gating and strategy used to differentiate GFP and RFP under micafungin treatment. [file mbio.01180-23-s0005.tif]

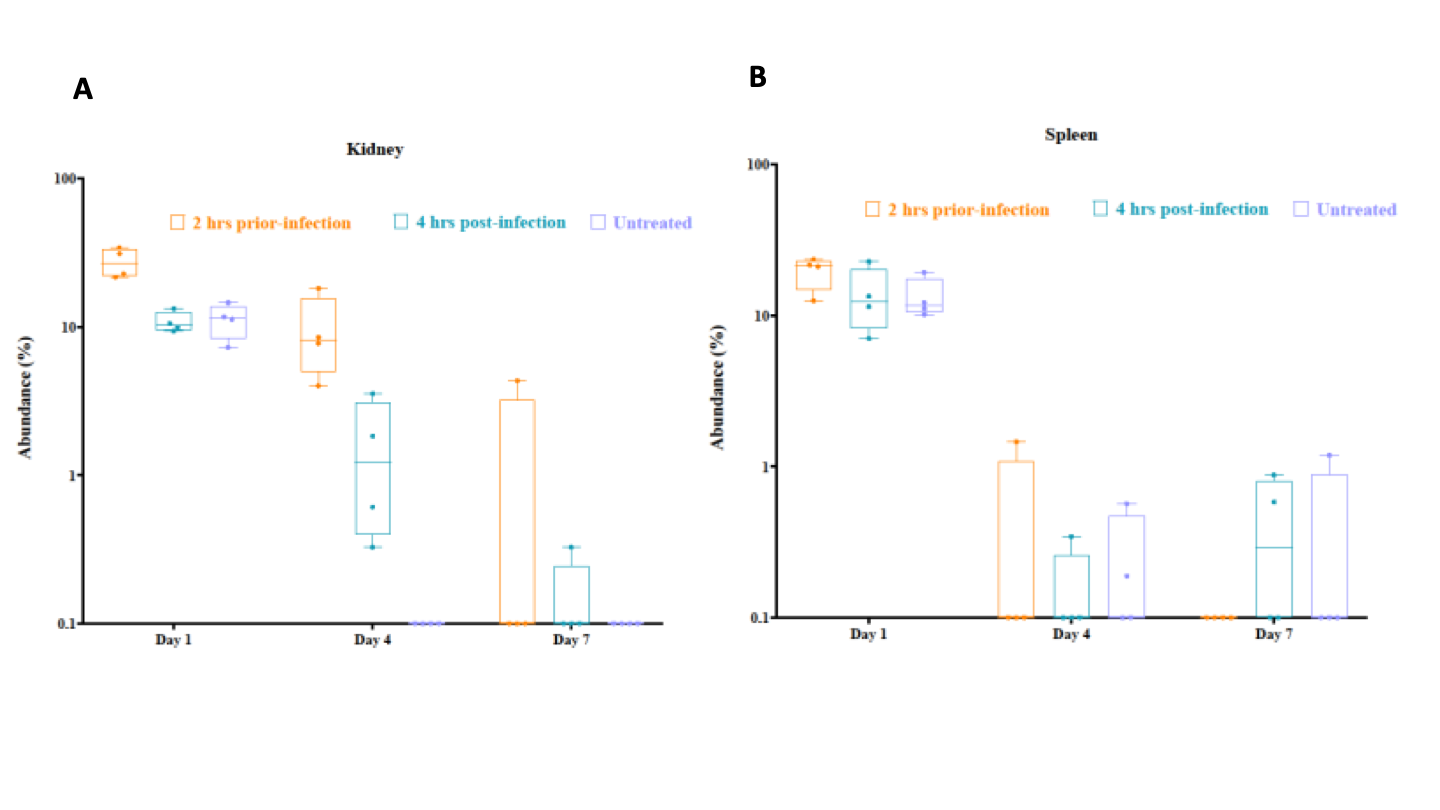

Supplement: Figure S6 — Mice infected with the petite isolate BYP41 had a lower burden, especially in the kidney at early time points, compared to mice treated with a humanized dosage of caspofungin. [file mbio.01180-23-s0006.tif]
