# Supplementary material for: Overlooked Candida glabrata petites are echinocandin tolerant, induce host inflammatory responses, and display poor in vivo fitness
Source: mBio. 2023 Sep 29;14(5):e01180-23. doi: 10.1128/mbio.01180-23 (PMC10653939; doi:10.1128/mbio.01180-23)
Supplement: Supplemental Legends — Legends for supplemental figures. [file mbio.01180-23-s0007.pdf]

Figure S1. Describes the processes involved in the development of laboratory and clinically derived petite *C. glabrata* isolates. The petite mutant isolates recovered from CBS138 were obtained under the selection pressure of fluconazole (A). BYP41 was derived from an immunocompetent patient after fluconazole treatment and was genetically close to BYP40 (B). Supplementation with certain metabolites fosters the growth rate of petite isolates. Petites grow poorly on yeast-nitrogen-based (YNB) media, reflecting their defective mitochondria and their inability to assimilate non-fermentable carbon sources. To determine the metabolic deficiencies of *C. glabrata* petite strains, we measured their growth rates in yeast nitrogen base (YNB) medium and YNB individually supplemented with arginine (20 mg/L), leucine (60 mg/L), glutamine (2 mM), glutamate (5 mM), histidine (20 mg/L), lysine (60 mg/L), aspartate (20 mg/L), menadione (5 μg/ml), thymidine (100 μg/ml), adenine (20 mg/L), and hemin (1 μg/ml). Consistent with this, BYP41 and C5, D5, and F2 were more similar in their metabolite dependency profiles, whereas G5 and DPL248 clustered with non-petite isolates. The addition of leucine, arginine, and glutamine significantly improved the growth rate of petite isolates (C). As expected, petites grew slowly in unsupplemented YNB, with G5 and DPL248 growing better than BYP41, C5, D5, and F2 but still more slowly than non-petite strains (D).

Figure S2. Mitochondrial DNA (mtDNA) coverage of petite and non-petite isolates using whole-genome sequencing identified that petite isolates have a lower mtDNA than non-petites, with BYP41 having the lowest mtDNA content (A). Similar to petite isolates, the *Rdm9Δ* isolate was resistant to fluconazole (B). Similar to petite isolates, *Rdm9Δ* overexpresses efflux pumps and *PDR1* compared to the parental strain CBS138 (C).

Figure S3. Similar to petite isolates, *Rdm9Δ* has a significantly lower level of ATP than the parental strain CBS138 (A). Similar to petite isolates, *Rdm9Δ* has a lower mitochondrial membrane potential than the parental strain CBS138 (B). Similar to petite isolates, *Rdm9Δ* shows poor growth on YNB (C), and supplementation with specific metabolites ameliorates its growth (D). Similar to petite isolates, the *Rdm9Δ* isolate does not grow inside macrophages.

Figure S4. Non-responsiveness of intracellular petites to echinocandins. Unlike non-petite parental isolates, petites are not efficiently killed by echinocandins upon engulfment by macrophages (A). Petite isolates have a higher echinocandin tolerance to various echinocandin concentrations once incubated in RPMI (B).

Figure S5. Flow cytometry gating and strategy used to differentiate GFP and RFP under micafungin treatment. Similar to petite isolates, intracellular *rdm9Δ* is extremely tolerant to micafungin.

Figure S6. Mice infected with the petite isolate BYP41 had a lower burden, especially in the kidney at early timepoints, compared to mice treated with a humanized dosage of caspofungin (A). The treated arm included mice that received caspofungin 2 hrs prior to infection or 4 hrs post-infection (B).
